# Infrastructure-health nexus in Brazil: a scoping review

**DOI:** 10.1186/s41256-025-00441-x

**Published:** 2025-09-02

**Authors:** Flavio Pinheiro Martins, Carol Vigurs, Mariana Matera Veras, Lusi Morhayim, Monica Lakhanpaul, Priti Parikh

**Affiliations:** 1 Engineering for International Development Centre, The Bartlett School of Sustainable Construction, UCL Faculty of the Built Environment, 1-19 Torrington Place, London, WC1E 7HB UK; 2 Social Research Institute, University College, 18 Woburn Square, London, WC1H 0NR UK; 3 Present Address: Laboratory of Experimental Air Pollution (LIM05), Department of Pathology, School of Medicine, University of São Paulo, 455 Dr. Arnaldo Avenue, São Paulo, 01246 903 Brazil; 4 The Bartlett School of Sustainable Construction, UCL Faculty of the Built Environment, 1-19 Torrington Place, London, WC1E 7HB UK; 5 UCL - Great Ormond Street Institute of Child Health, University College London, 30 Guildford Street, London, WC1N 1EH UK

**Keywords:** Health infrastructure, Population health, Scoping review, Brazil, Health system, SUS, Public health policy, Healthcare access

## Abstract

**Background:**

Health system development requires robust infrastructure systems support, particularly in countries with significant regional and socioeconomic disparities. Brazil’s experience with its Unified Health System offers important insights into how the infrastructure and built environment is linked to health outcomes especially in underserved populations. This scoping review examines how different infrastructure systems such as sanitation, transportation, educational facilities, housing, influence population health in Brazil through two key pathways: (1) their role in shaping environmental conditions that affect health, and (2) their impact on healthcare service delivery among vulnerable populations.

**Methods:**

Following PRISMA-ScR checklist, we conducted a systematic search of studies published between 2013-2024 across Scopus, Web of Science, and PubMed databases. Search terms included infrastructure systems (sanitation, transportation, housing, educational facilities), health outcomes (universal health coverage, infectious diseases, maternal health), and population descriptors (vulnerable, indigenous, underserved) combined with Brazil-specific terms. Inclusion criteria focused on studies examining physical infrastructure's impact on health outcomes in underserved Brazilian communities, published in English or Portuguese. After applying exclusion criteria including publication year restrictions, language filters, geographic limitations, duplicate removal, and non-article format exclusions, 68 studies met inclusion criteria following screening and quality assessment using the Critical Appraisal Skills Programme (CASP) checklist. Our analysis applied an infrastructure framework examining institutional, personal, and material infrastructure dimensions. Data extraction captured infrastructure systems, healthcare service tiers (primary, secondary, tertiary), and specific health outcomes. Synthesis involved thematic analysis to identify patterns in infrastructure-health relationships, revealing three interconnected dimensions that form the Infrastructure-Health Nexus framework.

**Results:**

The study revealed three interconnected dimensions of infrastructure impact: Supporting Health & Wellbeing, Service Access and Delivery, and Community Engagement. This framework shows how sanitation, transportation, educational, housing, and waste management systems affect health outcomes, with underserved populations facing particular challenges. Healthcare workforce programs serve as interim solutions, with educational facilities simultaneously functioning as health hubs for service delivery and community engagement. The study highlights misalignment between infrastructure investment and UHC objectives.

**Conclusions:**

The Infrastructure-Health Nexus framework, building on Buhr’s complementarity concept, shows how infrastructure shapes health outcomes through pathways requiring integrated planning. While current research focuses predominantly on primary care aspects, Brazil’s epidemiological transition calls for broader health system considerations, suggesting reconceptualization of infrastructure systems planning as integral to health system development.

**Supplementary Information:**

The online version contains supplementary material available at 10.1186/s41256-025-00441-x.

## Introduction

Universal Health Coverage (UHC) aims to ensure all individuals receive needed healthcare without financial hardship, addressing disparities and improving public health [1, 2]. Brazil’s Unified Health System (SUS) provides universal coverage to approximately 200 million people. Established in the 1988 Federal Constitution, the SUS operates through three fundamental principles: (1) universality in access, establishing health as a citizenship right; (2) equity, ensuring fair resource distribution; and (3) integrality, integrating health promotion, disease prevention, treatment, and rehabilitation [3]. Despite these principles, the system faces significant challenges. Fragmented governance and gaps in coordination between federal, state, and municipal levels affect service delivery efficiency, with public health financing comprising only 41% of total expenditures [4]. These constraints were exacerbated by Constitutional Amendment 95 (EC95) in 2016, which limited federal health expenditure for 20 years, with projected losses of approximately US$93 billion by 2036 [3, 5]. This underfunding exacerbates existing weaknesses in the healthcare facilities network, including insufficient Intensive Care Unit (ICU) beds and uneven facility distribution [6, 7]. These limitations are not isolated; they are fundamentally linked to Brazil’s broader infrastructure conditions.

Infrastructure influences health outcomes through multiple pathways, impacting both service delivery and population health. Brazil’s development demonstrates this: improvements in basic sanitation between 1990 and 2015 were linked to significant reductions in infant mortality (from 53.4 to 14.0 per 1,000 live births) [6]. However, persistent inequalities continue to shape health outcomes. As of 2021, only 55.8% of the population had access to public sewage networks, with Black and Indigenous populations bearing a disproportionate burden—representing 47% of those lacking sanitation [8]. These infrastructure gaps create compounding effects: inadequate environmental conditions foster communicable diseases, while limited transportation restricts healthcare access [9]. According to the Organisation for Economic Co-operation and Development (OECD), Brazil’s geographic inequities in health outcomes are stark, with higher infant mortality rates in the North and Northeast. Furthermore, inefficient hospital and healthcare service distribution exacerbate these disparities, with lower-income and rural communities facing systemic barriers to quality care. This cycle of disadvantage highlights how infrastructure deficits simultaneously elevate health risks and limit care access, perpetuating inequities, particularly for vulnerable populations [4].

**Fig. 1:**
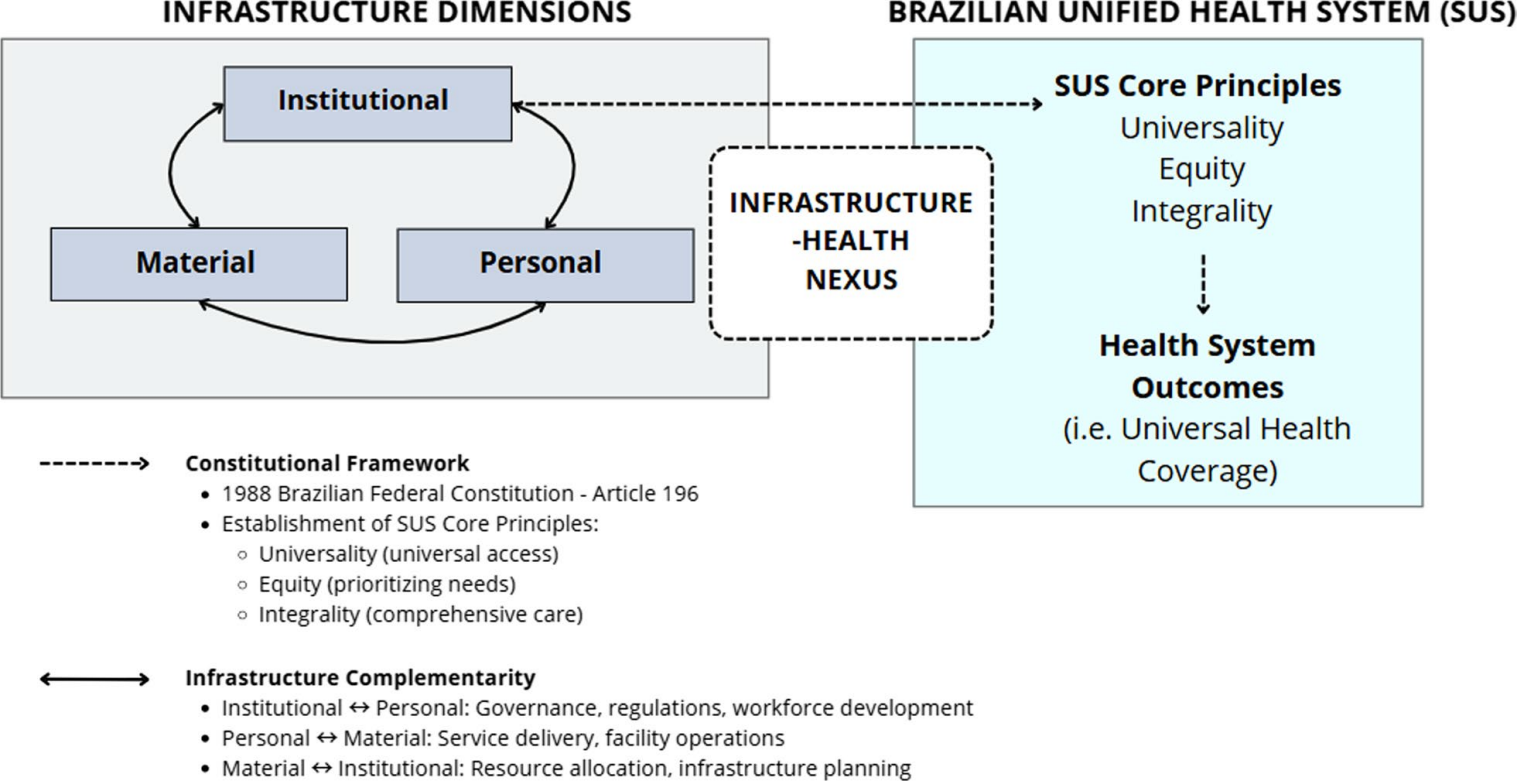
Infrastructure Dimensions and their Relationship with Brazil’s Unified Health System. *Legend* Framework showing the three interconnected infrastructure dimensions (institutional, personal, and material) that support Brazil’s SUS operations and influence health outcomes. *Source* Developed by authors

To address these challenges, understanding the SUS operational framework is critical. The system serves approximately 200 million people, embodying health as a constitutional right and requiring comprehensive state action. The health system operates through interconnected elements—from foundational rules [10] to the physical infrastructure that enables it. These elements can be understood as institutional, personal, and material dimensions supporting health outcomes [11]. Figure 1 illustrates how these elements interact within Brazil’s SUS. In practice, they work together daily: community health agents implement programs while navigating complex environments. Healthcare delivery takes place within a broader infrastructure network (transportation, education, energy, water, sanitation, housing, and waste management)—essential for fulfilling Brazil’s constitutional commitment to universal healthcare.

While existing literature documents aspects of health-infrastructure relationships in Brazil, reviews have focused on specific domains: environmental health determinants [12], access barriers for indigenous communities [13], environmental exposure impacts [14], cultural adaptation of services [15], and climate-health relationships [16] (see Supplementary Data). These provide valuable insights, but a broader understanding of the links between infrastructure and health is needed. This review examines how infrastructure influences population health outcomes, particularly among vulnerable populations in Brazil. Through systematic analysis, we examine how infrastructure affects health and healthcare service delivery and impacts SUS goals. Building on infrastructure and health theoretical approaches [17, 18], our analysis revealed three interconnected infrastructure dimensions forming the Infrastructure-Health Nexus framework: (1) infrastructure supporting health and wellbeing, (2) infrastructure for healthcare services access and delivery, and (3) infrastructure for community engagement in health promotion and services.

## Methods

### Research design

This scoping review explores how infrastructure influences population health in underserved Brazilian communities, following Arksey and O’Malley’s methodological framework [19], which includes six stages: (1) identifying the research question, (2) identifying relevant literature, (3) selecting studies against predefined inclusion criteria, (4) charting the data, (5) collating, summarizing, and reporting the results, and (6) consultation with stakeholders. The reporting follows the PRISMA-ScR checklist [20]. The review timeframe (2013–2023) was selected to encompass critical challenges to the SUS, including Constitutional Amendment 95 which limited federal health expenditure [3, 5] and infrastructure deficiencies exposed during the pandemic [6, 7]. This 10-year period captures contemporary infrastructure-health relationships while ensuring sufficient literature volume for meaningful synthesis.

### Search strategy and information sources

We developed a comprehensive search strategy consistent with health system policy scoping reviews [21–23]. We conducted searches across three major databases: Scopus, Web of Science, and PubMed, selected for their complementary coverage across biomedical, social sciences, and interdisciplinary research. Search terms were developed through pearl-growing techniques by consulting relevant systematic reviews and scoping reviews, using controlled vocabulary and free-text terms in English and Portuguese. Our search strategy included key terms related to infrastructure systems (e.g., ‘sanitation’, ‘transportation’, ‘housing’, ‘educational facilities’), health outcomes (e.g., ‘universal health coverage’, ‘infectious diseases’, ‘maternal health’), and population descriptors (e.g., ‘vulnerable’, ‘indigenous’, ‘underserved’) combined with Brazil-specific geographic terms. The complete search strategy is provided in Supplementary Data.

While grey literature was not included in formal data extraction, key policy documents such as Organisation for Economic Co-operation and Development (OECD) reviews of health systems [4], Ministry of Health reports [6], and civil society assessments [8] were consulted to inform our research design and contextualize findings.

### Study selection and eligibility criteria

Inclusion criteria were structured using the PICOC framework:

- *P (Population)*: Underserved communities in Brazil (including vulnerable populations, indigenous communities, rural populations, urban slums dwellers, and other marginalized groups)
- *I (Intervention/Exposure)*: Physical infrastructure systems including transport, sanitation, housing, educational facilities, healthcare facilities, water systems, energy systems, and other built environment components
- *C (Comparison)*: No comparison required for the scoping review focus
- *(Outcome)*: Health outcomes, health coverage, health service access, population health indicators, or health-related quality of life measures
- *C (Context)*: Brazil, contemporary period (2013–2024)

The exclusion criteria comprised studies published more than 10 years ago (EC1), studies not published in English or Portuguese (EC2), studies conducted outside Brazil (EC3), systematic reviews, meta-analyses, editorials, and opinion pieces (EC4), and duplicate publications (EC5).

*Screening involved two stages*: title/abstract review followed by full-text review. The screening checklist was conducted by two reviewers (FM and MMV) who assessed a sample independently and then compared results. Disagreements in coding were resolved through discussion until consensus was reached. Boundary issues around scope or definition were discussed in regular meetings with an advisory group of experts in the field.

### Quality assessment

The quality of individual studies was assessed using the Critical Appraisal Skills Programme (CASP) [24] checklist, which assessed the reliability of the study findings based on how well the study described the research aims, methodology, design, data collection, and analysis rigor. A standardized form documented the process; the complete protocol and results are in the Supplementary Data.

### Data extraction and synthesis

Data were extracted between November 5–15, 2023 using a classification framework (see Supplementary Data), capturing key characteristics of the studies:

- *Infrastructure systems*: institutional, personal, and material
- *Health Service Tier*: primary, secondary, tertiary care
- *Health Focus Areas*: specific health outcomes studied
- *Theoretical frameworks*: conceptual approaches used

The data extraction checklist was conducted with two reviewers (FM and MMV) coding key characteristics of studies independently. Disagreements were resolved through discussion and we consulted a third reviewer as needed. Findings were synthesized through: (1) extraction of key relationships, (2) categorization of infrastructure-health connections, and (3) thematic analysis. This process revealed three interconnected dimensions characterizing the infrastructure-health nexus. The complete dataset (standardized XML format) is available on request.

## Results

**Fig. 2:**
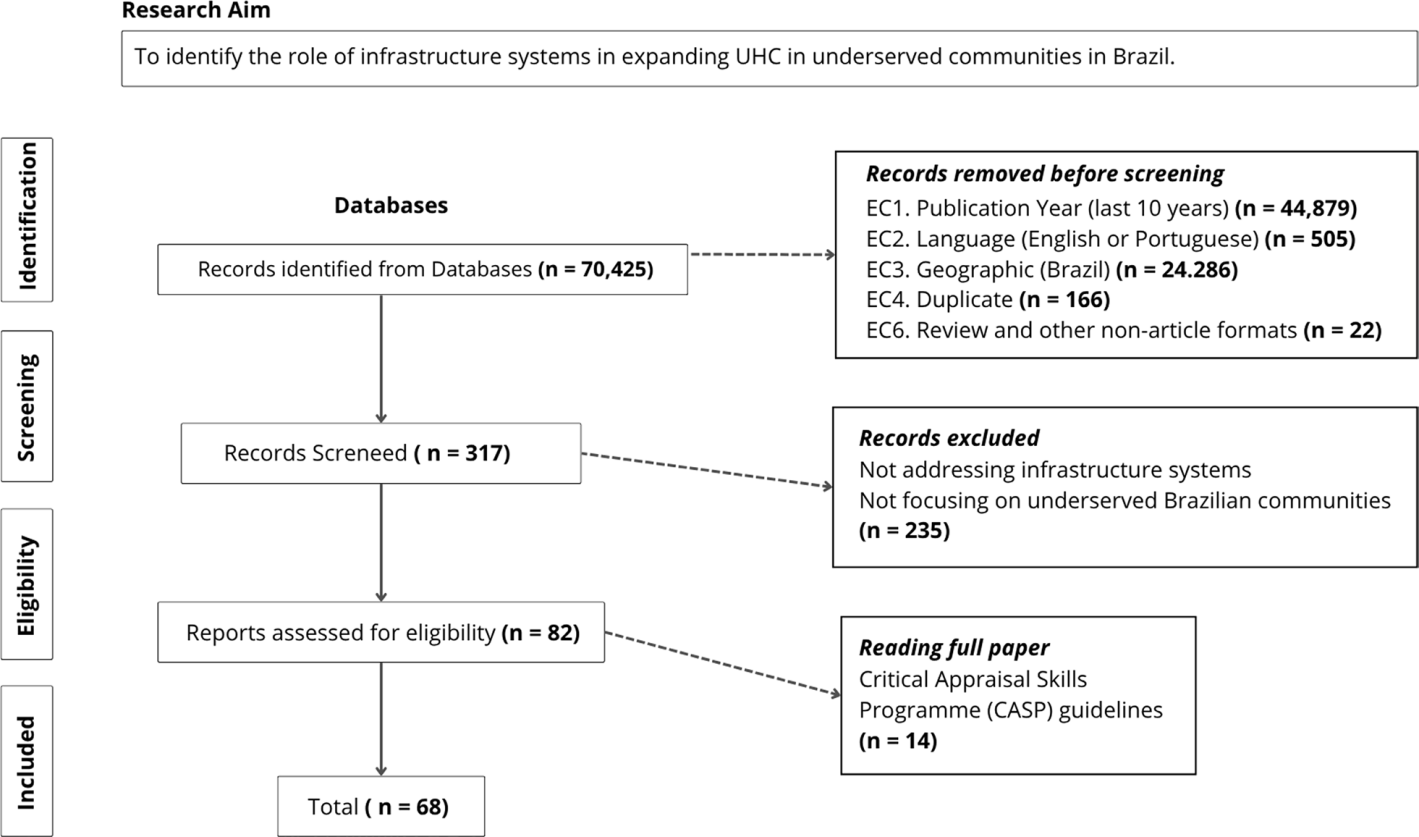
PRISMA flow diagram of study selection process. *Legend* Systematic selection process following PRISMA-ScR guidelines, from initial search of 70,425 records across three databases to final inclusion of 68 studies after screening and quality assessment

The search strategy identified 70,425 potentially relevant studies from Scopus (n = 47,027), Web of Science (n = 13,683), and PubMed (n = 9,715). Initial searches were conducted between November 5–15, 2023. After applying exclusion criteria including publication year restrictions (44,879 records), language filters (505 records), geographic limitations (24,286 records), duplicate removal (166 records), and non-article format exclusions (22 records), 317 records remained for title and abstract screening. After applying exclusion criteria during screening, 82 studies remained for full-text review. Following full-text assessment and quality evaluation, 68 studies met the inclusion criteria and were included in the final analysis (see Fig. 2).

### Study characteristics and evidence mapping

Following CASP quality assessment, 68 of 82 studies (83%) met inclusion criteria and demonstrated adequate methodological rigor, with 95% providing clear research aims, 88% using appropriate designs, and 90% presenting clear findings. Fourteen studies were excluded primarily for insufficient focus on infrastructure-health relationships or limited relevance to health equity.

Table 1 summarizes the key characteristics of included studies. The research shows concentrated activity in recent years (63.2% published 2020–2023), probably reflecting pandemic-era responsiveness. The field demonstrates alignment between infrastructure focus and health outcomes, with water/sanitation research predominantly addressing infectious diseases, while educational infrastructure spans multiple health domains.

**Table 1:** Characteristics of included studies

Characteristic	Category	n	%
Publication Year	2020–2023	43	63.2%
	2023 (peak year)	17	25.0%
	2013–2019	25	36.8%
Infrastructure System	Water & Sanitation	22	32.4%
	Educational facilities	21	30.9%
	Transportation	10	14.7%
	Housing	10	14.7%
	Waste management	5	7.4%
Health Focus Area	Communicable diseases	26	38.2%
	- Infectious diseases	22	32.4%
	- COVID-19	4	5.9%
	Maternal, child & reproductive health	13	19.1%
	Non-communicable diseases	12	17.6%
	Oral health	7	10.3%
Healthcare Service Level	Primary care only	56	82.4%
	Multi-level care	10	14.7%
	Secondary/tertiary only	2	2.9%

The emphasis on primary care settings (82.4%) reflects infrastructure’s fundamental role in community-level health delivery and prevention, aligning with the field’s commitment to addressing health equity through basic service systems rather than specialized medical care. Theoretical approaches were usually not explicitly stated but mostly relied on Social Determinants of Health frameworks, with some specific mentions of models such as One Health and Socio-Ecological approaches.

### Key patterns across the studies

Our analysis suggests how infrastructure systems influence population health through infrastructure conditions and healthcare service delivery, disproportionately burdening underserved communities. Indigenous communities face the greatest challenges from limited infrastructure, while urban informal settlements face distinct environmental hazards, and rural populations struggle with specialized healthcare service access. These vulnerabilities suggest marked racial disparities, particularly affecting Black and Indigenous populations [25–28]. Table 2 exemplifies how infrastructure relates to population health across Brazil’s diverse contexts. The evidence indicates complex relationships between infrastructure quality, population health, and social vulnerability, examined through the Infrastructure-Health Nexus framework: (1) supporting health and wellbeing, (2) enabling healthcare access and delivery, and (3) facilitating community engagement in health promotion and services.

**Table 2:** Examples of infrastructure systems and their impact on health focus areas

Study Title	Dimension	Infrastructure System	Health Focus Area	Main Findings
Mercury Exposure in Munduruku Indigenous Communities [25]	Infrastructure Supporting Health & Wellbeing	Sanitation and Water	Maternal, Child, and Reproductive Health	High mercury exposure (nearly 60% above critical levels) was found among Munduruku indigenous communities, with particular concerns for women of childbearing age and children [25]
Public health implications of particulate matter inside bus terminals in Sao Paulo [59]	Infrastructure Supporting Health & Wellbeing	Transportation	Non-Communicable Diseases	Bus commuters exposed to 94% higher PM10 doses inside terminals; high carcinogenic risk from chromium exposure
Socioeconomic inequities and hepatitis A virus infection in Western Brazilian Amazonian children [81]	Infrastructure Supporting Health & Wellbeing	Sanitation and Water	Infectious Diseases	32% of adolescents had at least one STI, with higher prevalence in areas with limited healthcare infrastructure
Geographic access to COVID-19 healthcare in Brazil [37]	Healthcare Services Access and Delivery	Healthcare Facilities	COVID-19	Substantial disparities in Intensive Care Unit (ICU) access during pandemic, particularly affecting black and poor communities in urban peripheries
Understanding how low-income communities gain access to healthcare services [48]	Healthcare Services Access and Delivery	Transportation	Multiple	Transport barriers create compound access challenges, particularly affecting emergency care access in rural regions
Treatment outcomes of tuberculosis patients in Brazilian prisons [82]	Healthcare Services Access and Delivery	Healthcare Facilities	Infectious Diseases	Higher rates of treatment default and poorer outcomes linked to inadequate prison healthcare infrastructure
School dental health education on oral hygiene status in Brazilian Quilombolas [57]	Infrastructure for Community Engagement	Educational Infrastructure	Oral Health	56.25% improvement in oral hygiene following school-based intervention; demonstrates effectiveness of educational facilities for health promotion
Family Health Strategy associated with increased dental visitation among preschool children [83]	Infrastructure for Community Engagement	Educational Infrastructure	Oral Health	Higher dental visitation rates (36% vs 23%) in areas with Family Health Strategy compared to traditional facilities
How, what, and why: housing, water & sanitation and wealth patterns in the Guarani Birth Cohort [84]	Infrastructure Supporting Health & Wellbeing	Housing	Maternal, Child, and Reproductive Health	Strong associations between substandard housing conditions and adverse health outcomes in indigenous communities
Educational technology on urinary tract infection for riverine pregnant women [85]	Infrastructure for Community Engagement	Educational Infrastructure	Maternal, Child, and Reproductive Health	Development of targeted educational materials improved health knowledge and practices in remote riverine communities

Examples are organized according to the Infrastructure-Health Nexus framework dimensions. Each study demonstrates specific pathways through which infrastructure systems influence health outcomes. The"Dimension"column categorizes studies according to our analytical framework, while"Main Findings"summarize key evidence on infrastructure-health relationships. Studies were selected to represent diverse geographic contexts, population groups, and infrastructure systems across Brazil

### Water, sanitation, and housing

Brazil’s sanitation infrastructure (water supply, sewage, solid waste, and drainage) is interwoven with public health outcomes. However, significant regional inequities in access and quality exist. Specifically, for water infrastructure, approximately 35 million Brazilians lack access to safely treated water, with only 57.5% of the population in northern regions having access [29]. Although intervention programs like PAT have shown promise in reducing under-five mortality and hospitalizations related to diarrhoea [30], nearly half the population still lacks complete sanitation [8]. This disparity is significant, as each US$1 invested in sanitation is estimated to save US$4.30 in health treatment costs [31]. These deficits create distinct community-level challenges. In urban informal settlements, residents face exposed sewage and contamination risks, with a significant portion lacking sewage connections and with untreated wastewater [32]. Conversely, remote communities, particularly Indigenous territories in the Amazon, often completely lack formal sanitation systems [25]. This limited access leads to increased infectious diseases and nutrition-related conditions like stunting [26, 33, 34]. Furthermore, the COVID-19 pandemic further highlighted these vulnerabilities, with higher infection and death rates in regions with poor sanitation [35, 36], reinforcing infrastructure quality as a mediator between location and health outcomes.

Similarly, housing conditions directly influence health: poor ventilation, overcrowding, inadequate materials, and poor drainage increase disease risks [37]. These factors are particularly pronounced in urban informal settlements, where housing density correlates with increased disease transmission [37, 38], whereas dispersed rural housing complicates healthcare access. Further highlighting these disparities, research on Indigenous Guarani and Quilombola communities shows strong links between housing conditions and adverse health outcomes, especially for maternal and child health [26, 27]. These communities face the added challenge with housing that fails to meet both traditional living patterns and basic health needs [39]. The most extreme manifestation of housing insecurity, homelessness, presents even more severe challenges, with the number of homeless families increasing dramatically from approximately 10,000 in 2012 to nearly 300,000 by 2024 [40]. A high percentage (68% to 85%) of the homeless population experiences mental health issues [41], creating a complex cycle of health deterioration linked to chronic pain, depression, and sleep disorders [42]. While both inadequate housing and homelessness impact health, homeless individuals face added mobility challenges during emergencies [43]. Despite targeted services, the lack of stable housing remains a fundamental barrier to healthcare access and positive outcomes [44]. Ultimately, these inequities demonstrate how socioeconomic factors, mediated by housing quality (or its absence), shape health outcomes.

### Healthcare facilities and workforce programs

Healthcare service infrastructure in Brazil exhibits distinct regional patterns reflecting historical development inequities, with striking contrasts in healthcare equipment distribution—from a 700% excess supply of MRI units in the Federal District to significant shortages in four Northeastern states [45]. In informal urban settlements, socioeconomic disparities create complex barriers to healthcare access, where factors like education level, family income, and area of residence significantly impact access [46, 47]. Across different regions, secondary and tertiary healthcare access faces particular challenges, including proximity/remoteness of facilities, walking safety, public transport inadequacies, personal security risks, and poor healthcare service quality [48]. Infrastructure limitations further compound these challenges, many regions failing to meet the Brazilian Ministry of Health’s minimum requirements for essential equipment like dialysis machines, hospital beds, and bone densitometers, particularly affecting municipalities beyond a 50 km radius of major healthcare centers [45]. Alternative solutions like river transport in Amazonian regions and telehealth networks attempt to bridge these gaps, though implementation faces significant maintenance and operational challenges, especially in indigenous communities where cultural and infrastructural barriers intersect [49].

Addressing some of these limitations, the More Doctors Program, created in 2013, emerges as a significant response through workforce deployment. The program deployed over 14,000 physicians to more than 3,800 municipalities, with 77.7% to priority and vulnerable areas [50]. Studies document improvements in service delivery, including increases of 5.9% in medical appointments, 9.4% in consultations, and 29.7% in home visits [51]. The program’s impact extended to health outcomes, with significant reductions in hospitalizations for ambulatory care-sensitive conditions and improved continuity of care [52]. User satisfaction surveys indicated enhanced quality of care, characterized by more dedicated consultation time, attentive listening, and detailed physical examinations [53]. The program reduced the number of municipalities experiencing physician shortages from 1,200 to 777, demonstrating its success in addressing human resource gaps in primary care despite persistent physical infrastructure limitations [54].

### Schools networks

In the reviewed studies, schools appear as platforms for healthcare service delivery and promotion, serving multiple health functions, from hosting vaccination programs (which, for instance, increased HPV vaccine coverage from 16.1% to 50.5%) to providing spaces for health education and screening [55]. Program effectiveness demonstrates regional variation. Urban schools capitalize on superior infrastructure and resource availability, contrasting sharply with rural institutions that struggle to maintain basic facilities. Indigenous and traditional communities represent a unique context where schools function as cultural intermediaries, enabling the synthesis of traditional health practices with modern healthcare approaches [56]. Studies demonstrate the successful implementation of school-based interventions: oral health programs significantly improved hygiene status among Quilombola children, with 56.25% showing better outcomes [57], though the scalability of such culturally-specific interventions across Brazil’s diverse contexts remains limited by infrastructure disparities between well-resourced urban and under-resourced rural schools. Similarly, multicomponent interventions promoted physical activity and reduced computer use among adolescents in low HDI areas [58].

### Transportation

Transportation networks serve as critical determinants of healthcare service access across Brazil’s diverse regions. Pereira et al. [37] documented social and spatial inequalities during the COVID-19 pandemic, revealing challenges across varied geographic contexts. Urban peripheries face overcrowded public transport and long commutes to specialized facilities, whereas rural communities often lack basic transport infrastructure entirely. Indigenous territories and remote areas face particularly severe barriers, where limited medical infrastructure and transportation severely impact emergency healthcare delivery [46]. These mobility-related disparities particularly affect low-income communities across all regions, creating a self-reinforcing cycle where access barriers exacerbate existing health vulnerabilities [48]. Beyond these access challenges, transportation systems also create direct health risks through environmental exposure. Studies in São Paulo reveal multiple health impacts: public transport creates direct health risks through exposure to black carbon and toxic metals like chromium inside bus terminals, leading to intolerable carcinogenic risks [59], reflecting broader urban planning approaches that prioritize operational efficiency over health protection for the low-income populations who depend most heavily on public transport. Traffic density and NO2 concentrations correlate with increased respiratory cancer, particularly in areas with lowest socioeconomic status [58].

### Infrastructure-health nexus framework

**Fig. 3:**
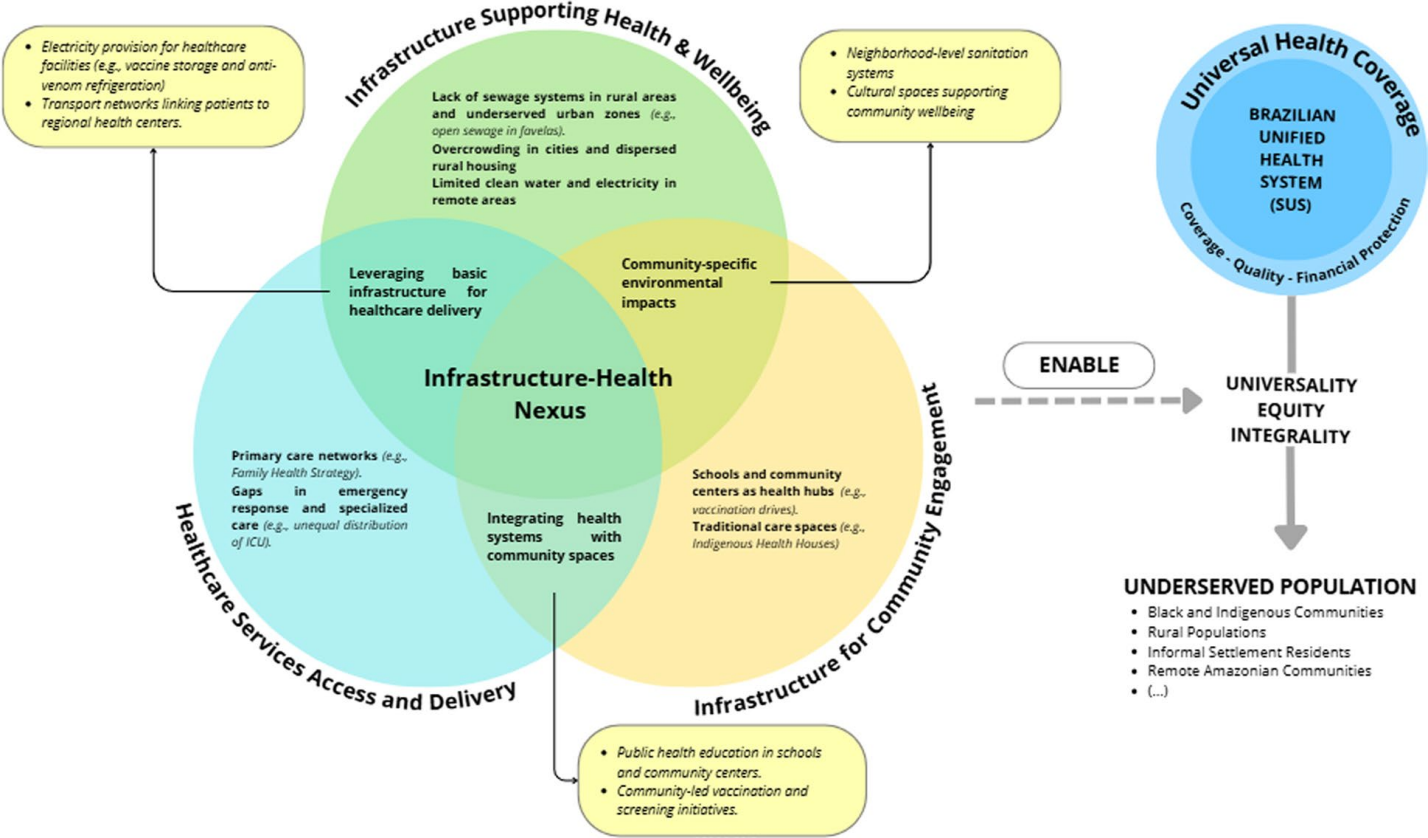
Infrastructure-Health Nexus in Brazil. *Legend* Conceptual framework showing three interconnected dimensions: Supporting Health & Wellbeing, Healthcare Services Access and Delivery, and Community Engagement, and their influence on population health outcomes in Brazil. *Source* Developed by the authors

The Infrastructure-Health Nexus framework emerged from our synthesis and analysis of the 68 reviewed studies, revealing how infrastructure shapes health outcomes in Brazil. Our analysis identified three interconnected dimensions that collectively organize these relationships: (1) Supporting Health & Wellbeing (fundamental conditions impacting health through sanitation, housing, and utilities); (2) Healthcare Services Access and Delivery (quality of facilities, services, and transportation networks); and (3) Community Engagement (spaces and structures enabling community participation). Although existing literature documents various infrastructure-health connections, our framework (Fig. 3) provides a novel structure for understanding these relationships.

## Discussion

### Infrastructure supporting health & wellbeing

The apparent disconnect between physical infrastructure development and constitutionally guaranteed health rights in Brazil suggests systemic challenges. Although the constitution envisions health as a fundamental right, Brazil’s infrastructure investment has fallen to under 2% of GDP, below the 5–7% of comparable economies [60]. This underinvestment affects multiple sectors, with investment patterns favoring urban centers. The sanitation sector demonstrates these patterns: federal budget execution decreased from US$74.5 million in 2014 to US$24.4 million in 2023, contributing to regional disparities. In contrast, 91.6% of the population in the South has access to safe water services, only 64.2% do in the North [6], indicating how infrastructure investment correlates with socioeconomic status.

Federal initiatives have attempted to address these gaps through programs like the Growth Acceleration Program (PAC), launched in 2007, which coordinates interventions across transportation, housing, and basic services. The program’s Water for All component has allocated US$1.03 billion for 540 municipalities (2023–2026), with additional initiatives in electrification and social housing [61]. However, implementation faces limitations due to coordination gaps between federal, state, and municipal governments. These patterns reflect the inherently contested nature of resource allocation in health systems, where competing beliefs and priorities about infrastructure investments fundamentally shape health outcomes [18]. This weakness becomes evident during political transitions, as shown by the 95% reduction in social housing funding from US$196.44 million to US$13.48 million between 2022–2023 [62]. These fluctuations highlight how institutional guarantees often fail to translate into sustained material improvements, revealing the gap between policy objectives and implementation.

These infrastructure limitations appear to intersect with Brazil’s epidemiological and demographic transition, as the healthcare system must address both infectious diseases and chronic non-communicable diseases (NCDs). Concurrent infectious disease burdens persist, with Brazil recording 583,960 new cases of neglected tropical diseases during 2016–2020, predominantly in areas with limited infrastructure [63]. Dengue fever represents a significant public health challenge, particularly in urban settings where inadequate waste management and water storage support mosquitoes [64]. In underserved regions, infrastructure deficiencies affect both infectious disease control and NCD care access. Evidence suggests that the link between infrastructure deficits and health outcomes particularly affects indigenous and traditional populations, pointing to a possible misalignment between infrastructure investment and the SUS’s constitutional mandate for equitable health provision.

### Healthcare services access and delivery

Furthermore, access to healthcare services in Brazil’s SUS is fundamentally shaped by transport networks—universal coverage depends on the population’s ability to reach service points. Regional disparities are evident in both transportation and facility distribution, creating distinct challenges. Urban areas face congestion and facility overload, conversely rural regions struggle with basic connectivity and limited transportation options, such as roads and waterways, which are often inadequate [65]. These challenges are compounded by unreliable or out-of-reach electricity in isolated areas, impacting essential services from vaccination programs to facility operations. The inequality in healthcare infrastructure is exemplified in the diverse data on spatial distribution [66], with a notable example being the excess of MRI availability of 700% in the Brazilian capital [45], reflecting the misalignment between health equity goals and the means to achieve them.

To address these structural limitations, the Brazilian SUS has leveraged infrastructure complementarity [11] – categorizing health human resources as a human dimension of infrastructure. This approach exemplifies the distinction between"hard"physical infrastructure and"social"infrastructure that maintains services and societal standards, with Brazil’s community health worker model representing a strategic emphasis on soft infrastructure to compensate for hard infrastructure limitations [17]. Due to limited facility availability, human resources have become central to service delivery. The community health workers network and the More Doctors Program (2013) demonstrate how healthcare professionals can supplement material infrastructure limitations in vulnerable regions. Both programs have contributed to reduced hospitalizations and improved primary care delivery [50, 51]. The More Doctors program reached nearly 4,000 municipalities (out of 5,565) serving approximately 63 million Brazilians. Hone et al. (2020) found that the program increased primary care doctor density by 15.1 per 100,000 population and achieved significant health improvements, including annual reductions in amenable mortality of 1.06 per 100,000 [67]. Analysis shows improved health coverage and reduced preventable hospitalizations in participating municipalities [68]. However, the program faced operational challenges, including lower doctor deployment to priority municipalities than intended [67], exacerbated during the 2018 political transition when the withdrawal of Cuban doctors affected medical coverage, correlating with an increase in preventable deaths [68, 69].

Though human resource programs have shown benefits, the rising burden of non-communicable diseases presents additional challenges, as their management requires specialized facilities and equipment that healthcare professionals alone cannot provide [3, 7]. In this context, telemedicine serves as a material infrastructure solution to address gaps in specialist care. Programs such as eConsults and telecardiology have shown potential to improve access to specialized healthcare, particularly in rural and underserved areas. By utilizing telecom and IT systems, telemedicine facilitates referral efficiency and provides essential services like remote monitoring and consultations, helping address workforce and infrastructure limitations [70, 71]. This experience suggests how personnel infrastructure may temporarily bridge, but likely cannot permanently resolve, physical environment infrastructure gaps in healthcare delivery, demonstrating the interplay between institutional, personal, and material infrastructure dimensions [11].

### Infrastructure for community engagement

Finally, Brazil’s Federal Constitution mandates community participation in the health system through health councils and conferences, structured with 50% community members, 25% providers, and 25% health system managers [4]. The Family Health Strategy implements this framework through multidisciplinary primary care teams, including community health workers, who deliver services from acute care to health promotion and chronic disease management [6]. These councils serve as deliberative bodies, proposing strategies and monitoring health policy implementation across municipal and state levels. The Indigenous Health Support Centers (CASAIs) provide the physical infrastructure for this engagement, offering care support and accommodation while bridging traditional and conventional healthcare practices. However, research shows that institutional protocols can inadvertently reinforce power asymmetries when standardized procedures override cultural sensitivities in care delivery [72].

Brazil’s school-based HPV vaccination campaigns demonstrate how this participatory framework operates in practice. These initiatives integrate public health policies with educational institutions, mobilizing healthcare workers, school staff, and parents and simultaneously using schools as vaccination sites. This approach increased vaccination coverage from 16.1% to 50.5% [73]. By leveraging existing educational infrastructure for HPV prevention, these initiatives reduce future demands on specialized healthcare services for cervical cancer treatment, demonstrating how basic infrastructure can support both preventive care and long-term health system sustainability. Beyond improving vaccine accessibility, the integration enhanced health education and built community trust, particularly addressing regional disparities in healthcare delivery [74]. The success of these campaigns shows how aligned institutional policies, healthcare workers, and community facilities can effectively meet diverse population health needs while maintaining cultural sensitivity.

### Theory and policy implications

The Infrastructure-Health Nexus framework presented here is inspired by infrastructure theory [9–11] and emerging research on infrastructure-health connections [17] to provide a different lens on health system development. The Brazilian case presents a distinctive example of infrastructure complementarity, where investments in community health workers and family health teams have achieved significant health outcomes despite physical infrastructure limitations.

The framework provides policy opportunities for aligning infrastructure investment with Universal Health Coverage objectives. Regional disparities in healthcare equipment distribution demonstrate the need for cross-sectoral coordination mechanisms that mandate health impact assessments for major infrastructure decisions. Targeted investment programs should prioritize social housing and sanitation funding in vulnerable communities to address existing inequities. Programs like More Doctors and school-based health interventions demonstrate effective approaches to leveraging existing infrastructure through human resources and community engagement rather than building new facilities, positioning health and wellbeing as fundamental societal needs [75].

This infrastructure complementarity from the SUS, presented here, might address policy and material gaps in fragmented health systems globally. For instance, South Africa and Mexico experience unequal healthcare access among specific population groups despite similar constitutional health guarantees [76, 77]. Indonesia’s public–private dynamics where private care predominantly serves high-income populations [78] also reflect challenges within Brazil’s dual SUS and supplementary health systems. Beyond mirroring some barriers, countries develop complementary solutions: South Africa’s alternative medication distribution through pick-up points effectively relieves pressure on facilities [79], exemplifying infrastructure optimization that aligns with Brazil’s patient-centered CHW model. Another example of potential learning between universal healthcare models is the National Health Service piloting Brazilian community-based approaches in London boroughs [80], suggesting growing interest in the personal infrastructure-centered UHC approach.

### Limitations and future research directions

This review encompasses 68 studies, representing substantial literature for a single country. However, this should not be interpreted as an exhaustive evidence base but rather as revealing the current state of research and indicating dimensions for further investigation. Our broad scope across multiple infrastructure systems and diverse populations may limit analytical depth, yet this comprehensive approach highlights interconnected challenges and patterns potentially missed in narrower studies. Our focus on three databases and intentional avoidance of broad"Infrastructure"AND"Health"search terms may have excluded relevant sources, particularly grey literature from non-academic stakeholders that could capture implementation experiences and community perspectives.

Future research should examine how infrastructure planning can better align with health outcomes, particularly in supporting both primary and specialized healthcare service access. Government databases and NGO monitoring reports could provide supplementary data on infrastructure investment patterns and health outcomes, particularly for tracking policy implementation effectiveness and community-level impacts. Specific priority areas include water-energy-health systems in Amazonian communities, transportation-education-health integration in traditional settlements, and climate-resilient infrastructure development. This integration is crucial for advancing UHC in contexts marked by regional and socioeconomic disparities, recognizing that infrastructure and health policy are mutually reinforcing domains that both fundamentally concern the public interest [75].

## Conclusions

Our analysis of infrastructure systems in Brazil’s pursuit of UHC shows both effective adaptations and ongoing challenges. The health system has developed practical solutions to physical infrastructure constraints, from community health workers navigating informal settlements to schools serving as healthcare service platforms. However, these adaptations, despite being innovative, operate within persistent structural limitations. The evidence suggests that Brazil’s development approach, which often prioritizes operational efficiency, results in solutions that address immediate needs but may not resolve underlying access barriers. As Brazil experiences its epidemiological transition, the disconnect between infrastructure planning and health system requirements becomes more evident. Understanding these dynamics requires appreciation of the inherently political nature of infrastructure development and health policy processes, where competing priorities and resource allocation decisions fundamentally shape health outcomes. Future research should examine how infrastructure planning can better align with health outcomes, particularly in supporting both primary and specialized healthcare service access. This integration is crucial for advancing UHC in contexts marked by regional and socioeconomic disparities, recognizing that infrastructure and health policy are mutually reinforcing domains that both fundamentally concern the public interest.

## Supplementary Information

Supplementary file 1.

## Data Availability

The complete dataset (standardized XML format) is available on request to the corresponding author.

## References

[1] World Health Organization, The World Bank. Tracking universal health coverage. global monitoring report. Geneva: World Health Organization; 2023. p. 2023.

[2] United Nations. Sustainable Development Goals: 3. Good health and well-being. https://www.un.org/sustainabledevelopment/health/; 2015 [accessed 12 October 2024].

[3] Massuda A, Dall’Alba R, Chioro A, Temporão JG, Castro MC. After a far-right government: challenges for Brazil’s unified health system. Lancet. 2023;401(10380):886–8. 36841254 10.1016/S0140-6736(23)00352-5

[4] OECD. Oecd reviews of health systems: Brazil 2021. Paris: OECD Publishing; 2021.

[5] Camargo KR Jr, Azevedo e Silva G, Giovanella L. Brazil’s national health care system at risk for losing its universal character. Am J Public Health. 2020;110(6):811–2. 32374681 10.2105/AJPH.2020.305649 PMC7204477

[6] Castro MC, Massuda A, Almeida G, Menezes-Filho NA, Andrade MV, de Souza Noronha KVM, Atun R. Brazil’s unified health system: the first 30 years and prospects for the future. Lancet. 2019;394(10195):345–56. 31303318 10.1016/S0140-6736(19)31243-7

[7] Paschoalotto MAC, Lazzari EA, Castro MC, Rocha R, Massuda A. The health systems resilience: notes for a research agenda for the SUS. Saude Debate. 2023;46:156–70.

[8] Civil Society Working Group for the 2030 Agenda (CSWG 2030A). 2030 Agenda for Sustainable Development: Spotlight Report 2023 Brazil Synthesis. https://gtagenda2030.org.br/relatorios-luz/; 2023 [accessed 12 October 2024].

[9] Garnelo L, Parente R, Puchiarelli M, Correia P, Torres M, Herkrath F. Barriers to access and organization of primary health care services for rural riverside populations in the Amazon. Int J Equity Health. 2020. 10.1186/s12939-020-01171-x. 32731874 10.1186/s12939-020-01171-x PMC7394681

[10] Jochimsen R. Theorie der Infrastruktur: Grundlagen der marktwirtschaftlichen Entwicklung [Theory of Infrastructure: Foundations of Market Economy Development]. J.C.B: Mohr; 1966.

[11] Buhr W. What is infrastructure? Discussion Paper No. 107–03. Department of Economics, School of Economic Disciplines, University of Siegen; 2003.

[12] Rojas-Rueda D, Vaught E, Buss D. Why a new research agenda on green spaces and health is needed in Latin America: results of a systematic review. Int J Environ Res Public Health. 2021;18(11): 5839. 34072319 10.3390/ijerph18115839 PMC8198896

[13] Sandes LFF, Freitas DA, de Souza MFNS, de Sousa Leite KB. Primary health care for South-American indigenous peoples: an integrative review of the literature. Rev Panam Salud Publica. 2018;42(1):e163. 31093191 10.26633/RPSP.2018.163 PMC6385845

[14] Dórea JG, Marques RC. Mercury levels and human health in the Amazon basin. Ann Hum Biol. 2016;43(4):349–59. 27230737 10.1080/03014460.2016.1192682

[15] Oliveira MVG, Abreu MM, Welch JR, Coimbra CE Jr. Coping with hypertension among indigenous peoples in Brazil and the role of the primary care nurse: A critical review from a transcultural perspective. Nurs Rep. 2021;11(4):942–54. 34968280 10.3390/nursrep11040086 PMC8715468

[16] Fernandez-Guzman D, Lavarello R, Yglesias-González M, Hartinger SM, Rojas-Rueda D. A scoping review of the health co-benefits of climate mitigation strategies in South America. Lancet Reg Health Am. 2023. 10.1016/j.lana.2023.100602. 37876667 10.1016/j.lana.2023.100602 PMC10593577

[17] Harris P, De Leeuw E. Infrastructure and health: laying down the big connections for well-being. Oxford Open Infrastructure and Health. 2022;1(1):1–7.

[18] De Leeuw E, Harris P, Kim J, Yashadhana A. A health political science for health promotion. Glob Health Promot. 2021;28(4):17–25. 34510937 10.1177/17579759211034418

[19] Levac D, Colquhoun H, O’brien KK. Scoping studies: advancing the methodology. Implement Sci. 2010;5:1–9. 20854677 10.1186/1748-5908-5-69 PMC2954944

[20] Tricco AC, Lillie E, Zarin W, O’Brien KK, Colquhoun H, Levac D, Moher D, Peters MDJ, Horsley T, Weeks L, Hempel S, Akl EA, Chang C, McGowan J, Stewart L, Hartling L, Aldcroft A, Wilson MG, Garritty C, Lewin S, Godfrey CM, Macdonald MT, Langlois EV, Soares-Weiser K, Moriarty J, Clifford T, Tunçalp Ö, Straus SE. PRISMA Extension for Scoping Reviews (PRISMA-ScR): Checklist and Explanation. Ann Intern Med. 2018. 10.7326/M18-0850 30178033

[21] Shittu E, Lakhanpaul M, Vigurs C, Sarkar K, Koch M, Parikh P, Campos LC. A rapid systematic scoping review of research on the impacts of water contaminated by chemicals on very young children. Sci Total Environ. 2023. 10.1016/j.scitotenv.2023.164604. 37271388 10.1016/j.scitotenv.2023.164604

[22] Mengistu TS, Khatri R, Erku D, Assefa Y. Successes and challenges of primary health care in Australia: a scoping review and comparative analysis. J Glob Health. 2023. 10.7189/jogh.13.04043. 37387471 10.7189/jogh.13.04043 PMC10311945

[23] Ahmed ST, Haider SS, Hanif S, Anwar HB, Mehjabeen S, Closser S, Sarker M. A scoping review on integrated health campaigns for immunization in low-and middle-income countries. Health Policy Plan. 2023;38(10):1198–224. 37699072 10.1093/heapol/czad082 PMC10752386

[24] Critical Appraisal Skills Programme (CASP). CASP Qualitative Studies Checklist. CASP; 2018. Available from: https://casp-uk.net/wp-content/uploads/2018/03/CASP-Qualitative-Checklist-2018_fillable_form.pdf.

[25] Basta PC, Vega CM, Vasquez OC, Cartaxo SJM, Oliveira RA, Carneiro FFR, et al. Mercury exposure in Munduruku indigenous communities from Brazilian Amazon: Methodological background and an overview of the principal results. Int J Environ Res Public Health. 2021;18(17):9222. 34501811 10.3390/ijerph18179222 PMC8430525

[26] Leite MS, Cardoso AM, Coimbra CE, Welch JR, Gugelmin SA, Lira PCI, Escobar AL. Prevalence of anemia and associated factors among indigenous children in Brazil: results from the first national survey of indigenous people’s health and nutrition. Nutr J. 2013;12:1–11. 23714275 10.1186/1475-2891-12-69 PMC3681561

[27] Santarém VA, Doline FR, Ferreira IB, Farinhas JH, Biondo LM, de Souza Filho RT, Biondo AW. One health approach to toxocariasis in Brazilian indigenous populations, their dogs, and soil contamination. Front Public Health. 2023;11:1220001. 37744519 10.3389/fpubh.2023.1220001 PMC10517057

[28] Rocha HA, Correia LL, Campos JS, Silva AC, Andrade FO, Silveira DI, Cunha AJ. Factors associated with non-vaccination against measles in northeastern Brazil: clues about causes of the 2015 outbreak. Vaccine. 2015;33(38):4969–74. 26215369 10.1016/j.vaccine.2015.07.027

[29] Gargiulo AH, Duarte SG, Campos GZ, Landgraf M, Franco BDG, Pinto UM. Food safety issues related to eating in and eating out. Microorganisms. 2022;10(11):2118. 36363709 10.3390/microorganisms10112118 PMC9695559

[30] Rasella D. Impact of the water for all program (PAT) on childhood morbidity and mortality from diarrhea in the Bahia state, Brazil. Cad Saude Publica. 2013;29:40–50. 23370023 10.1590/s0102-311x2013000100006

[31] Cavalcanti A, Teixeira AA, Pontes KV. Evaluation of the efficiency of basic sanitation integrated management in Brazilian municipalities. Int J Environ Res Public Health. 2020;17(24): 9244. 33321908 10.3390/ijerph17249244 PMC7764331

[32] Kligerman DC, OliveiraCardoso TA, Cohen SC, Azevedo DCB, Toledo GA, Azevedo APCB, Charlesworth SM. Methodology for a comprehensive health impact assessment in water supply and sanitation programmes for Brazil. Int J Environ Res Public Health. 2022;19(19):12776. 36232082 10.3390/ijerph191912776 PMC9565092

[33] Lima FS, Scalize PS, Gabriel EFM, Gomes RP, Gama AR, Demoliner M, et al. Escherichia coli, Species C Human Adenovirus, and Enterovirus in Water Samples Consumed in Rural Areas of Goiás, Brazil. Food Environ Virol. 2021;1–12. 10.1007/s12560-021-09504-x 34792781

[34] Mata MM, Santana ABC, Martins FP, Medeiros MAT. Quality and access to water for human consumption: a look at the state of Amazonas, Brazil. Cien Saude Colet. 2024;29(8): e05442023. 39140536 10.1590/1413-81232024298.05442023

[35] Vasconcelos IMP, Muÿlder CFD. The institutional voids, sanitation and water deficits and the first covid-19 numbers in brazil. Rev Adm UFSM. 2021;14:1221–38.

[36] Diep L, Martins FP, Campos LC, Hofmann P, Tomei J, Lakhanpaul M, Parikh P. Linkages between sanitation and the sustainable development goals: A case study of Brazil. Sustain Dev. 2021;29(2):339–52.

[37] Pereira RH, Braga CKV, Servo LM, Serra B, Amaral P, Gouveia N, Paez A. Geographic access to COVID-19 healthcare in Brazil using a balanced float catchment area approach. Soc Sci Med. 2021;273: 113773. 33609968 10.1016/j.socscimed.2021.113773 PMC7879934

[38] Dickin SK, Schuster-Wallace CJ. Assessing changing vulnerability to dengue in northeastern Brazil using a water-associated disease index approach. Glob Environ Change. 2014;29:155–64.

[39] Cardoso AM, Resende PC, Paixao ES, Tavares FG, Farias YN, Barreto CTG, Siqueira MM. Investigation of an outbreak of acute respiratory disease in an indigenous village in Brazil: contribution of Influenza A (H1N1) pdm09 and human respiratory syncytial viruses. PLoS ONE. 2019;14(7): e0218925. 31283762 10.1371/journal.pone.0218925 PMC6613774

[40] Ministry of Social Development. Total homeless families registered in the Single Registry. VIS DATA 3. https://aplicacoes.cidadania.gov.br/vis/data3/; 2024 [accessed 12 October 2024].

[41] Menezes IRA, Uchida RR, Tenorio DS, Araujo JEB, Lima NNR, Lopes GCD, Neto MLR. The helplessness and invisibility of the mental health of homeless people in Brazil. Lancet Reg Health Am. 2022;15: 100368. 36778070 10.1016/j.lana.2022.100368 PMC9903991

[42] Braga NT, Brito LS, Garcia JBS. Homeless individuals and their vulnerability to pain, depression, and sleep: Narrative review. BrJP. 2024;7: e20240042.

[43] do Couto AC, Gravinatti ML, Pellizzaro M, Kmetiuk LB, Yamakawa AC, da Silva EC, et al. One health approach on serosurvey of anti-*Leptospira* spp. in homeless persons and their dogs in South Brazil. One Health. 2022;15: 100421. 36277102 10.1016/j.onehlt.2022.100421 PMC9582539

[44] Bombonatti GR, Saidel MGB, Rocha FM, Santos DS. Street clinics and the healthcare of vulnerable homeless communities in Brazil: a qualitative study. Int J Environ Res Public Health. 2022;19(5): 2573. 35270266 10.3390/ijerph19052573 PMC8910102

[45] Amaral P, Rocha TAH, Barbosa ACQ, Lein A, Vissoci JRN. Spatially balanced provision of health equipment: a cross-sectional study oriented to the identification of challenges to access promotion. Int J Equity Health. 2017. 10.1186/s12939-017-0704-x. 29202757 10.1186/s12939-017-0704-x PMC5715625

[46] Murta F, Strand E, de Farias AS, Rocha F, Santos AC, Rondon EAT, et al. “Two Cultures in Favor of a Dying Patient”: Experiences of Health Care Professionals Providing Snakebite Care to Indigenous Peoples in the Brazilian Amazon. Toxins. 2023;15(3):194. 36977085 10.3390/toxins15030194 PMC10051728

[47] Zolnikov TR, Cruvinel V, Lopez P, Pezeshkian F, Stoves-Tucker L, Galato D, et al. A qualitative study on noncommunicable diseases in waste pickers in Brazil. J Health Pollut. 2021;11(30): 210603. 34267990 10.5696/2156-9614-11.30.210603 PMC8276723

[48] Guimarães T, Lucas K, Timms P. Understanding how low-income communities gain access to healthcare services: A qualitative study in São Paulo. Brazil J Transp Health. 2019;15: 100658.

[49] Oliveira JPA, Sanchez MN, Santos LMP. The mais médicos (more doctors) program: the placement of physicians in priority municipalities in Brazil from 2013 to 2014. Cien Saude Colet. 2016;21(9):2719–27. 27653057 10.1590/1413-81232015219.17702016

[50] Mattos E, Mazetto D. Assessing the impact of more doctors’ program on healthcare indicators in Brazil. World Dev. 2019;123: 104617.

[51] Fontes LFC, Conceição OC, Jacinto PDA. Evaluating the impact of physicians’ provision on primary healthcare: evidence from Brazil’s more doctors program. Health Econ. 2018;27(8):1284–99. 29770534 10.1002/hec.3775

[52] Comes Y, Trindade JS, Shimizu HE, Hamann EM, Bargioni F, Ramirez L, Santos LMP. Evaluation of user satisfaction and service responsiveness in municipalities enrolled in the Mais Médicos (more doctors) program. Cien Saude Colet. 2016;21:2749–59. 27653060 10.1590/1413-81232015219.16202016

[53] Girardi SN, Van Stralen ACS, Cella JN, Wan Der Maas L, Carvalho CL, Faria EO. Impact of the Mais Médicos (More doctors) program in reducing physician shortage in Brazilian primary healthcare. Cien Saude Colet. 2016;21(9):2675–84. 27653053 10.1590/1413-81232015219.16032016

[54] Teixeira JC, Vianna MSC, Vale DB, Arbore DM, Perini THW, Couto TJT, et al. School-based HPV vaccination: the challenges in a Brazilian initiative. Rev Bras Ginecol Obstet. 2021;43(12):926–31. 34933386 10.1055/s-0041-1740279 PMC10183876

[55] Jardim CPT, Dias JM, Grande AJ, Veras AB, Ferri ÉK, Quadros FA, Harding S. Co-developing a health promotion programme for indigenous youths in Brazil: a concept mapping report. PLoS ONE. 2023;18(2): e0269653. 36791063 10.1371/journal.pone.0269653 PMC9931109

[56] Barbosa Filho VC, Bandeira ADS, Minatto G, Linard JG, Silva JAD, Costa RMD, et al. Effect of a multicomponent intervention on lifestyle factors among Brazilian adolescents from low human development index areas: a cluster-randomized controlled trial. Int J Environ Res Public Health. 2019;16(2):267. 30669291 10.3390/ijerph16020267 PMC6352556

[57] Lima IAB, da Silva ME, Barasuol AM, Almeida RJL, dos Santos Figueiredo FW, da Silva GA, Quaresma FRP. School dental health education on oral hygiene status in Brazilian Quilombolas: a prospective study. Int J Health Promot Educ. 2024;62(1):53–62.

[58] Ribeiro AG, Downward GS, de Freitas CU, Neto FC, Cardoso MRA, de Oliveira MDRD, et al. Incidence and mortality for respiratory cancer and traffic-related air pollution in São Paulo. Brazil Environ Res. 2019;170:243–51. 30594696 10.1016/j.envres.2018.12.034

[59] Nogueira T, Kumar P, Nardocci A, de Fatima Andrade M. Public health implications of particulate matter inside bus terminals in Sao Paulo, Brazil. Sci Total Environ. 2020;711: 135064. 31831243 10.1016/j.scitotenv.2019.135064

[60] Raiser M, Clarke R, Procee P, Briceno-Garmendia C, Kikoni E, Kizito J, Viñuela L. Back to planning: How to close Brazil’s infrastructure gap in times of austerity. World Bank; 2017.

[61] Casa Civil. Novo PAC: Água para Todos. Presidência da República. https://www.gov.br/casacivil/pt-br/novopac/agua-para-todos; 2024.

[62] Doca G. Bolsonaro corta 93% da verba do Casa Verde Amarela em 2023, e não há dinheiro nem para concluir obras paradas. O Globo. 2022 Sep 16.

[63] Ministry of Health. Epidemiological Bulletin: Morbidity, mortality, and national response in the context of the Sustainable Development Goals 2016–2020 - Neglected Tropical Diseases in Brazil. Secretariat of Health Surveillance and Environment. https://www.gov.br/saude/pt-br; 2024 [accessed 12 October 2024].

[64] Kawahama FH, Santos LBL, Cirilo PR, Souza LF, Macau EEN. Minimum vector control intensity to get a stable fixed point in a mosquito dynamic model. Trends Comput Appl Math. 2023;24(3):521–33.

[65] Science Panel for the Amazon. A new infrastructure for the Amazon: Policy brief. United Nations Sustainable Development Solutions Network; 2023.

[66] Ranzani OT, Bastos LSL, Gelli JGM, Marchesi JF, Baião F, Hamacher S, Bozza FA. Characterisation of the first 250,000 hospital admissions for COVID-19 in Brazil: A retrospective analysis of nationwide data. Lancet Respir Med. 2021;9(4):407–18. 33460571 10.1016/S2213-2600(20)30560-9 PMC7834889

[67] Hone T, Powell-Jackson T, Santos LMP, de Sousa MF, de Oliveira FP, Bertolini RF, Millett C. Impact of the Programa Mais médicos (more doctors Programme) on primary care doctor supply and amenable mortality: quasi-experimental study of 5565 Brazilian municipalities. BMC Health Serv Res. 2020;20(1):873. 32933503 10.1186/s12913-020-05716-2 PMC7491024

[68] Pires B. Anais do descalabro I: Bolsonaro desidratou Mais Médicos e pôs no lugar um ninho de falcatruas. Piauí Magazine. 2023 Jun.

[69] Pinto HA. O que tornou o Mais Médicos possível? Análise da entrada na agenda governamental e da formulação do Programa Mais Médicos [Doctoral dissertation]. Federal University of Rio Grande do Sul; 2021.

[70] Moraes JL, Paixão GMM, Gomes PR, Mendes EMAM, Ribeiro ALP, Beda A. A novel algorithm to assess the quality of 12-lead ECG recordings: validation in a real telecardiology application. Physiol Meas. 2023;44(3): 035006. 10.1088/1361-6579/acbc09 36896841

[71] Catapan SC, Bruckmann G, Nilson LG, Caffery LJ, Kelly JT, Calvo MCM, Boing AF. Increasing primary care capacity and referral efficiency: a case study of a telehealth centre econsult service in brazil. J Telemed Telecare. 2024. 10.1177/1357633X241235426 38446874

[72] Ribeiro AA, Arantes CIS, Gualda DMR, Rossi LA. Historical and cultural aspects of the provision of care at an indigenous healthcare service facility. Cien Saude Colet. 2017;22:2003–12. 28614519 10.1590/1413-81232017226.13362016

[73] Fregnani JHTG, Carvalho AL, Eluf-Neto J, Ribeiro KCB, Kuil LM, Silva TA, Villa LL. A school-based human papillomavirus vaccination program in Barretos, Brazil: final results of a demonstrative study. PLoS ONE. 2013;8(4): e62647. 23638130 10.1371/journal.pone.0062647 PMC3634818

[74] Chiang EDDO, Baker ML, Figueroa-Downing D, Baggio ML, Villa LL, Neto JE, Evans DP. “Those who love, vaccinate”: parental perceptions of hpv vaccination. J Hum Growth Dev. 2015;25(3):341.

[75] Harris P, Riley E, Dawson A, Friel S, Lawson K. “Stop talking around projects and talk about solutions”: Positioning health within infrastructure policy to achieve the sustainable development goals. Health Policy. 2020;124(6):591–8. 30545623 10.1016/j.healthpol.2018.11.013

[76] White JA, Rispel LC. Policy exclusion or confusion? Perspectives on universal health coverage for migrants and refugees in South Africa. Health Policy Plan. 2021;36(8):1292–306. 33848339 10.1093/heapol/czab038 PMC8428584

[77] Gómez-Dantés O, Flamand L, Cerecero-García D, Morales-Vazquez M, Serván-Mori E. Origin, impacts, and potential solutions to the fragmentation of the Mexican health system: a consultation with key actors. Health Res Policy Syst. 2023;21(1):80. 37525130 10.1186/s12961-023-01025-2 PMC10388521

[78] Septiono W. Equity challenges in Indonesian health care. Lancet Glob Health. 2023;11(5):e646–7. 37061303 10.1016/S2214-109X(23)00110-9

[79] Mash R, Christian C, Chigwanda RV. Alternative mechanisms for delivery of medication in South Africa: A scoping review. S Afr Fam Pract. 2021;63(1): a5274. 10.4102/safp.v63i1.5274 PMC8424755 34476963

[80] Donnelly L, Marquis C. The NHS is close to collapse – could a radical scheme from Brazil’s favelas save it? The Telegraph. 2025 Apr 7.

[81] Mantovani SA, Delfino BM, Martins AC, Oliart-Guzmán H, Pereira TM, Branco FL, et al. Socioeconomic inequities and hepatitis A virus infection in Western Brazilian Amazonian children: spatial distribution and associated factors. BMC Infect Dis. 2015;15:1–12. 26471064 10.1186/s12879-015-1164-9 PMC4608050

[82] Ribeiro Macedo L, Reis-Santos B, Riley LW, Maciel EL. Treatment outcomes of tuberculosis patients in Brazilian prisons: a polytomous regression analysis. Int J Tuberc Lung Dis. 2013;17(11):1427–34. 24125446 10.5588/ijtld.12.0918

[83] Feldens CA, Fortuna MJ, Kramer PF, Ardenghi TM, Vítolo MR, Chaffee BW. Family health strategy associated with increased dental visitation among preschool children in Brazil. Int J Paediatr Dent. 2018;28(6):624–32. 30175414 10.1111/ipd.12421 PMC6188830

[84] Caldas ADR, Nobre AA, Brickley E, Alexander N, Werneck GL, Farias YN, Cardoso AM. How, what, and why: housing, water & sanitation and wealth patterns in a cross-sectional study of the Guarani Birth Cohort, the first Indigenous birth cohort in Brazil. Lancet Reg Health Am. 2023. 10.1016/j.lana.2023.100496. 37214221 10.1016/j.lana.2023.100496 PMC10193232

[85] Neves PVT, Rodrigues ILA, Pereira AA, Andrade EGRD, Nogueira LMV, Maia RP, Moraes CMDS. Educational technology on urinary tract infection for riverine pregnant women: shared construction. Cogitare Enfermagem. 2023;28: e87352.

