# Supplementary material for: Infrastructure-health nexus in Brazil: a scoping review
Source: Glob Health Res Policy. 2025 Sep 2;10:42. doi: 10.1186/s41256-025-00441-x (PMC12403449; doi:10.1186/s41256-025-00441-x)
Supplement: Supplementary file 1 [file 41256_2025_441_MOESM1_ESM.docx]

**APPENDIX I - ADDITIONAL REVIEW DATA**

Search query components defined according to the PICOC framework

| **PICOC Elements*** | **Description** | **Number of keywords** | **Key Examples** |
| --- | --- | --- | --- |
| Intervention (I) | "infrastructure" - terms related to infrastructure improvements | 107 | Transportation infrastructure, Water and sanitation, Educational facilities, Housing infrastructure, Energy infrastructure, Health facilities, Digital infrastructure, Green infrastructure |
| Outcome (O) | "Universal Health Coverage" - health outcomes influenced by infrastructure | 81 | Universal Health Coverage, Maternal Health, Communicable Diseases, Non-communicable Diseases, Mental Health, Environmental Health, Health Access, Health Equity |
| Population (P) | "underserved" - specifying the target demographic | 19 | Vulnerable, Indigenous, Traditional, Riverine, Marginalized populations, Disadvantaged groups, Quilombola, Rural populations |
| Context (C) | (brazil* OR brasil*) - geographical focus of the research | 2 | Brazil, Brasil |
| - | PUBYEAR > 2013 AND PUBYEAR < 2024 - publication year range for recent research | - | - |
| - | AND (LIMIT-TO (LANGUAGE, "English") OR LIMIT-TO (LANGUAGE, "Portuguese")) - language filter | - | - |

*Note: The Comparison element was excluded as this scoping review aimed to map existing literature rather than compare specific interventions or approaches.

**Full Search query**
Detailed list of the 107 keywords related to "Intervention," the 81 keywords to "Outcome", and the 19 to "population" can be found in the supplementary material.

| **(TITLE-ABS-KEY (**  "Infrastructure and Engineering" OR "Urban Infrastructure" OR "Rural Infrastructure" OR "Transportation Infrastructure" OR "Energy Infrastructure" OR "Communication and Information Technology Infrastructure" OR "Water and Sanitation Infrastructure" OR "health Infrastructure" OR "Educational Infrastructure" OR "Social Infrastructure" OR "Environmental Infrastructure" OR "Physical Infrastructure" OR "Digital Infrastructure" OR "Industrial Infrastructure" OR "Public Infrastructure" OR "Private Infrastructure" OR "Critical Infrastructure" OR "Resilient Infrastructure" OR "Built environment" OR "Wash" OR "Water, sanitation and hygiene" OR "Water and sanitation" OR "Water, sanitation and health" OR "Water and hygiene" OR "Sanitation and hygiene" OR "Drinking water" OR "Sanitation facilities" OR "Hygiene practices" OR "Water treatment" OR "Wastewater management" OR "Water supply" OR "Water quality" OR "Sewage systems" OR "Toilet facilities" OR "Open defecation" OR "Handwashing" OR "Environmental Health" OR "Public health engineering" OR "Water safety" OR "Water scarcity" OR "Water insecurity" OR "Water pollution" OR "Sanitation access" OR "Hygiene education" OR "Community water systems" OR "Safe water" OR "Waste management" OR "Waste disposal" OR "Waste treatment" OR "Waste collection" OR "Sanitary" OR "Solid waste" OR "Hazardous waste" OR "Municipal waste" OR "Industrial waste" OR "Electronic waste" OR "Agricultural waste" OR "Construction waste" OR "Waste-to-energy" OR "Waste segregation" OR "Waste reduction" OR "Recycling" OR "Waste auditing" OR "Waste characterization" OR "Waste assessment" OR "Waste remediation" OR "Waste stream" OR "Landfill" OR "Energy infrastructure" OR "Electric transmission" OR "Power lines" OR "Off-grid energy" OR "Natural gas" OR "Transportation infrastructure" OR "Public transportation" OR "Traffic monitoring" OR "Pollution Control" OR "Pollution Mitigation" OR "Pollution Prevention" OR "Pollution Reduction" OR "Pollution Management" OR "Air quality improvement" OR "Water quality improvement" OR "Emission reduction" OR "Contamination" OR "Urban illumination" OR "Green spaces" OR "Green infrastructure" OR "Sustainable infrastructure" OR "Blue infrastructure" OR "Land use" OR "Resilient cities" OR "Sustainable cities" OR "Natural infrastructure" OR "health facilities" OR "health infrastructure" OR "Medical infrastructure" OR "Medical facilities" OR "Schools" OR "Universities" OR "Facilities" OR "Colleges" OR "Higher education" OR "Housing infrastructure" OR "Social housing" OR "Public-sector housing" OR "Low-cost housing")  **AND**  **TITLE-ABS-KEY (**  "Universal health Coverage" OR "UHC" OR "Maternal Health" OR "Child Health" OR "Communicable Diseases" OR "AIDS" OR "HIV" OR "Tuberculosis" OR "Malaria" OR "Neglected Tropical Diseases" OR "NTDs" OR "Non-communicable Diseases" OR "NCDs" OR "Chronic Diseases" OR "Cardiovascular Diseases" OR "CVDs" OR "Diabetes" OR "Obesity" OR "Hypertension" OR "Neurodegenerative Diseases" OR "Alzheimer" OR "Parkinson" OR "Cancer" OR "Substance Use" OR "Drug Abuse" OR "Alcohol Abuse" OR "Addiction and Dependence" OR "Mental Health Disorders" OR "Road Injuries" OR "Traffic Accidents" OR "Sexual Health" OR "Reproductive Health" OR "Family Health" OR "Women's Health" OR "health Services" OR "Health Policy and Governance" OR "Community Health" OR "health Workers" OR "Environmental Pollution" OR "Contaminants" OR "Waterborne Diseases" OR "Foodborne Diseases" OR "Air Quality" OR "Medical Research" OR "health Development" OR "Vaccin*" OR "Immunization" OR "Tobacco Control" OR "Capacity Building for health" OR "Health Financing" OR "Training and Education" OR "health Workforce" OR "Nursing" OR "Medical Staff" OR "health Access" OR "Rural health" OR "Urban health" OR "health Disparities" OR "Telehealth" OR "Telemedicine" OR "Electronic Health Records" OR "EHR" OR "Health Insurance" OR "Public Health Programs" OR "Primary Care" OR "Preventive health" OR "Health Promotion" OR "Health Equity" OR "Health Outcomes" OR "health Quality" OR "Patient Safety" OR "health Costs" OR "Global Health" OR "Health Systems" OR "Health Infrastructure" OR "Health Research" OR "health Innovations" OR "Access to Medicines" OR "Health Technologies" OR "Health Data Analysis" OR "Health Surveillance" OR "Health Impact Assessment")  **AND**  **TITLE-ABS-KEY (**  "Vulnerable" OR "Traditional" OR "Maroon" OR "Indigenous" OR "First Nations" OR "Slaver*" OR "Riverine" OR "Marginalised populations" OR "Disadvantaged groups" OR "At-risk communities" OR "Underprivileged" OR "Socioeconomically challenged areas" OR "Historically oppressed groups" OR "Minority communities" OR "Socially excluded" OR "Underserved" OR "Impoverished" OR "Oppressed")  **AND**  **TITLE-ABS-KEY** ( brazil* OR brasil* )  )  AND ( PUBYEAR > 2013 AND PUBYEAR < 2024 )  AND ( LIMIT-TO ( LANGUAGE , "English" ) OR LIMIT-TO ( LANGUAGE , "Portuguese" ) ) |
| --- |

Classification structure for analysing infrastructure-health linkages

| **ID** | **Category** | **Predefined Codes** | **Reasoning** | **Operational Definition for coding** |
| --- | --- | --- | --- | --- |
| 1 | Infrastructure Systems | Transportation, Waste Management, Educational facilities, Sanitation and Water, Housing, Energy & Urban Development | Infrastructure here refers to the facilities or physical infrastructures, primarily public provisions, designed to fulfil human needs [9-11] | Physical infrastructures, primarily public provisions, designed to fulfill human needs. Coded based on primary infrastructure system addressed in each study |
| 2 | Healthcare Services | Primary, Secondary &  Tertiary | Informed by the Brazilian Unified Health System service tier | Service complexity levels within Brazil's SUS. Primary: community health, prevention; Secondary: specialized outpatient; Tertiary: high-complexity hospital care |
| 3 | Health Focus Areas | Chronic Diseases, Infectious Diseases, COVID-19,  Maternal, Child, and Reproductive Health, Nutrition-Related Conditions, Oral Health, Road Safety, Mental Health and Wellbeing & Substance Abuse | Keywords adapted from United Nations [2] Sustainable Development Goal 3 targets (Good Health and Wellbeing). | Health outcomes categorized according to UN SDG 3 targets, adapted for Brazilian epidemiological profile |
|  | **Category - Qualitative Descriptions** | | **Rationale/ Examples** | |
| 4 | Main Findings |  | Summarising the authors' key outputs from highlights, discussion, or conclusion. | |
| 5 | Research Gaps |  | Drawn from authors' limitations or future research agenda. | |
| 6 | Linkage Infrastructure-Health | | Analysing if the studies address this issue as a main target or as a background component of a broader health approach. | |
| 7 | Missing Links on Infrastructure-Healthcare | | Explicit or implicit calls for action in integrating infrastructure development and health outcomes. | |
| Note: Initial coding used predefined categories (Infrastructure Systems, Healthcare Services, Health Focus Areas) to organize the evidence. Through analysis of the coded data, three distinct dimensions of material infrastructure emerged: (1) Infrastructure Supporting Health & Wellbeing, (2) Infrastructure for Healthcare Services Access and Delivery, and (3) Infrastructure for Community Engagement. These dimensions form the organizational framework for presenting our findings in the Results section. | | | | |

**APPENDIX II - REVIEWS ADDRESSING UNDERSERVED POPULATIONS, HEALTH, AND INFRASTRUCTURE IN BRAZIL**

| **Author** | **Title** | **Scope** | **Review Design** | **Articles reviewed** | **Time frame** | **Missing Links Infrastructure-Health** |
| --- | --- | --- | --- | --- | --- | --- |
| Fonseca et al., (2013) | The vulnerabilities in childhood and adolescence and the Brazilian public policy intervention. | Reviews Brazilian policies helping children and teens facing various challenges. | Narrative review | N/A | 1990-  2012 | Discusses policy evaluation like the Statute of the Child and Adolescent and Bolsa Família, but lacks focus on the contribution of physical infrastructure like schools and sanitation, which are crucial for promoting health in underserved communities. |
| Dórea & Marques, (2016) | Mercury levels and human health in the Amazon Basin. | Focuses on studies examining mercury exposure and health outcomes among Amazonian populations | Narrative review | N/A | N/A | Provides insights into mercury's health effects but doesn't connect these to how health system deficiencies might exacerbate these impacts or limit access to interventions, particularly for vulnerable populations. |
| Asmus et al.,(2016) | A Systematic Review of Children’s Environmental  Health in Brazil | Examines the impact of environmental pollutants on children in Brazil. | Systematic review | 164 | N/A | Provides evidence of risks from pollutants but suggests more research is needed to explore how better infrastructure and access could mitigate these health risks in vulnerable communities. |
| Sandes et al., (2018) | Primary health care for South-American indigenous peoples: an integrative review of the literature | Investigates access to primary healthcare among indigenous groups in 8 South American countries. | Integrative review | 40 | 2007 - 2017 | Identifies several infrastructural barriers to healthcare access, such as transportation and cultural misunderstandings, highlighting deficiencies in health system infrastructure and resources, and underscoring the need for culturally competent service delivery. |
| Rojas-Rueda,Vaught, & Buss (2021) | A Systematic Review of Children's Environmental Health in Brazil | Studies the health benefits of green spaces in Latin America. | Systematic review | 19 | <sept. 2020 | While noting the benefits of green spaces, the review calls for more longitudinal studies on how infrastructure like schools and community centers can play a role in enhancing these benefits, especially for underserved communities. |
| Oliveira et al., (2021) | Coping with Hypertension among Indigenous Peoples in Brazil and the Role of the Primary Care Nurse: A Critical Review from a Transcultural Perspective | Reviews how primary care nurses manage hypertension among indigenous Brazilians. | Critical review | 17 | 2000 - 2020 | Points to systemic issues in health systems and nursing education as barriers to effective hypertension management among Indigenous peoples, indicating a need for further research on infrastructure improvements. |
| Fernandez-Guzman et al., (2023) | A scoping review of the health co-benefits of climate mitigation strategies in South America | Explores the health benefits of climate change strategies in South America. | Scoping review | 9 | 2001 - 2021 | Reviews various emission reduction strategies, but lacks a detailed examination of how these strategies interact with infrastructure to impact health outcomes in underserved areas. |
